# 2-Heptanol inhibits *Botrytis cinerea* by accelerating amino acid metabolism and retarding membrane transport

**DOI:** 10.3389/fpls.2024.1400164

**Published:** 2024-06-03

**Authors:** Fangfang Wu, Haibo Wang, Yankun Lin, Zesheng Qu, Bin Zheng, Shun Feng, Xinguo Li

**Affiliations:** School of Tropical Agriculture and Forest, National Key Laboratory of Tropcial Crop Breeding, Hainan University, Haikou, China

**Keywords:** gray mold, volatile organic compounds, antifungal mechanism, amino acid metabolism, membrane transport system

## Abstract

During the postharvest storage of tomatoes, they are susceptible to infection by *Botrytis cinerea*, leading to significant economic losses. This study evaluated the antifungal potential of 2-heptanol (2-HE), a volatile biogenic compound, against *B. cinerea* and explored the underlying antifungal mechanism. The results indicated that 2-HE effectively suppressed the growth of *B. cinerea* mycelia both *in vivo* and *in vitro* and stimulated the activities of antioxidative enzymes, including superoxide dismutase (SOD), peroxidase (POD), and catalase (CAT) in tomatoes. Furthermore, 2-HE reduced spore viability, compromised membrane integrity, and resulted in increased levels of extracellular nucleic acids, protein content, and membrane lipid peroxidation. Transcriptomic analysis revealed that 2-HE disrupted the membrane transport system and enhanced amino acid metabolism, which led to intracellular nutrient depletion and subsequent *B. cinerea* cell death. Additionally, the 2-HE treatment did not negatively impact the appearance or quality of the tomatoes. In conclusion, the findings of this study offer insights into the use of 2-HE as a biocontrol agent in food and agricultural applications.

## 1 Introduction

Postharvest decay of fruits and vegetables due to pathogen infection results in significant economic losses (Leng et al., 2022; Hosseini et al., 2023). *Botrytis cinerea* is a prevalent pathogen responsible for such decay, with the ability to infect over 200 plant species (Yu et al., 2018). Tomatoes, being rich in carotene and vitamin C, are one of the most vital agro-industrial crops (Szabo et al., 2021). The infection of *B. cinerea* is a primary factor impairing tomato quality postharvest (Williamson et al., 2007; Jiao et al., 2022). Currently, chemical fungicides are the most widespread agents used to control postharvest decay in fruits and vegetables (Chen and Ying, 2015). Nonetheless, the overuse of chemical fungicides has led to critical issues, including threats to food safety, environmental pollution, and increased resistance in fungi (Zhang X. et al., 2020; Zubrod et al., 2019). Consequently, there is an urgent need for safe biological antifungal agents to manage *B. cinerea* infections and mitigate the economic losses associated with postharvest tomatoes.

Recent research has highlighted the efficacy of bio-derived antifungal agents, deemed safe, in managing postharvest pathogens. Examples include magnolol (Cui et al., 2021), 3-methylthio-1-propanol (Feng et al., 2021), and curcumin (Hua et al., 2019). 2-Heptanol (2-HE), a volatile organic compound (VOC) produced by a range of microorganisms, plants, and animals (Du et al., 2022; Chang et al., 2023), is notable for its contribution to the aroma of gamma-aminobutyric acid white tea (Li et al., 2023) and has been identified in *Rubus coreanus* fruits (Yu et al., 2019), chicken sausages (Ozkara et al., 2019), and strains of *Aureobasidium pullulans* (Shi et al., 2023). In agriculture, 2-HE deters virulent green rice leafhoppers from colonizing rice plants infected with the rice dwarf virus (RDV) due to its repulsive properties (Chang et al., 2021). It also displays substantial repellent activity against pests such as *Tribolium castaneum* and *Rhyzopertha dominica* (Ukeh and Urnoetok, 2011). Moreover, 2-HE has demonstrated antifungal effects, for instance, reducing Fusarium crown and root rot incidence in tomato plants (Du et al., 2022), though its antifungal mechanisms merit further investigation. According to previous studies (Du et al., 2022; Shi et al., 2023), 2-HE has great biocontrol potential for controlling postharvest tomato diseases.

To our knowledge, the antifungal mechanisms of 2-HE have remained uncharacterized until now. This study employed both *in vitro* and *in vivo* experiments to assess the inhibitory effects of 2-HE on *B. cinerea*. The antifungal mechanisms underlying these effects were investigated using fluorescence microscopy, transcriptome analysis, and quantitative PCR (qPCR). The outcomes of this study will offer novel avenues for biological control and will serve as essential references for formulating ecological management strategies.

## 2 Materials and methods

### 2.1 Reagents, strains, and tomato

2-Heptanol (2-HE) (purity = 98%; CAS. No. 543–49-7) was commercially available from Macklin (Macklin Biochemical Co., Ltd., Shanghai, China). The fungal pathogen *B. cinerea* B05.10 was provided by Prof. Fangjie Xiong, Southwest University (Chongqing, China). Commercially mature tomatoes were harvested from Lingshui Farm (Hainan Province, China) and transported to the laboratory. Tomatoes with no damage and the same ripeness were cleaned with sterile water and then dried naturally for later use.

### 2.2 Antifungal activity of 2-HE against *B. cinerea in vitro*

The inhibitory activity of 2-HE on *B. cinerea* mycelium growth was evaluated using a non-contact assay. A *B. cinerea* mycelial plug (5-mm diameter) was placed on the side of the two-part plates containing potato dextrose agar medium, and 0, 5, 10, 15, and 20 µL of 2-HE were added to the other side of the two-part plates with filter paper (10 × 75 mm). The concentration conversion is shown in **Table 1** (Su and Liang, 2015). The 0 treatment was treated with an equal volume of distilled water instead of the maximum dose of the 2-HE treatment in the control group (0.21 μL cm^-3^). Then the dichotomous Petri dish was quickly sealed and cultured at 25°C for 5 days. The cross method was used to measure the colony diameter and calculate the inhibition rate. At each concentration, there are three biological replicates.

**Table 1:** Detailed concentration conversion.

Application amount (μL)	Petri dish volume (cm^3^)	Application concentration (μL cm^-3^)
0	95.4	0
5		0.05
10		0.10
15		0.16
20		0.21

### 2.3 Inhibitory efficacy of 2-HE against *B. cinerea in vivo*

Disease-free tomatoes of uniform size were cleansed with sterile distilled water and subsequently left to air-dry before further experimentation. After scraping the tomato equator with a sterile needle, the tomato wound was inoculated with a 3.0-mm *B. cinerea* mycelium plug. A Petri dish containing 2-HE at concentrations of 0 (distilled water as a control), 0.02, 0.05, 0.10, or 0.16 μL cm^-3^ was positioned at the base of a sealed glass chamber, with 20 inoculated tomatoes arranged on the perforated partition in the center of the chamber (Chen et al., 2022). After treatment, the diameter of the lesions on each tomato was recorded daily and sampled. During sampling, fruit tissue around the inoculation site (2 × 2 × 2 cm) was collected after removing the mycelium plug on the tomato surface. Each process was repeated three times.

### 2.4 Antioxidant enzyme activity and MDA content measurement

The samples collected in Section 2.3 were measured for antioxidant activity and malondialdehyde (MDA) content. Catalase (CAT), peroxidase (POD), superoxide dismutase (SOD) activity, and MDA content were determined by the CAT, POD, SOD, and MDA assay kit (Nanjing Jiancheng Bioengineering Institute, China). The test was performed according to the instructions of each kit, and the entire experiment was performed at 4°C. Each treatment was subjected to triplicate measurements to ensure experimental reliability.

### 2.5 Effects of 2-HE on tomato quality

To measure the effects of 2-HE on tomato quality, tomatoes with uniform size and maturity and without disease were washed and dried. A Petri dish containing 2-HE at concentrations of 0 (distilled water as a control), 0.02, 0.05, 0.10, or 0.16 μL cm^-3^ was positioned at the base of a sealed glass chamber, with 20 tomatoes arranged on the perforated partition in the center of the chamber (Chen et al., 2022). Tomatoes were collected on days 1, 2, 3, and 4 for the assessment of total soluble solids (TSS) and titratable acidity (TA). TSS content was quantified using a handheld refractometer (Atago, Japan), and the TA was ascertained through titration of tomato juice with a 0.01-M NaOH solution. Each treatment was conducted in triplicate.

### 2.6 Fluorescence microscopy

Cell viability and membrane integrity were assessed using fluorescein diacetate (FDA, Sigma) and propyl iodide (PI, Sigma) (Wang et al., 2020). A 10-µL spore suspension was spotted onto the inner surface of a Petri dish lid, followed by the introduction of varying 2-HE concentrations (0, 0.05, and 0.16 μL cm^-3^) to the base of the dish. The cultures were incubated at 25°C for 12 h. Post-incubation, spores were stained with FDA for 5 min and with PI for 10 min, both in darkness, at 25°C. The spores were then washed three times using phosphate-buffered saline (PBS) and examined under a fluorescence microscope (Zeiss, Germany). For each treatment, five random fields of view were chosen to quantify the number of stained spores.

### 2.7 MDA content and cellular inclusion leakage detection

Following a 5-day incubation of spores in potato dextrose broth at 25°C, 2.0 g of mycelium was harvested and resuspended in sterile water laced with 2-HE concentrations (0, 0.05, and 0.16 μL cm^-3^). After incubation for different times (0, 3, 6, 9, and 12 h), the supernatant and mycelium were collected, frozen with liquid nitrogen for 10 min, and then put into the refrigerator at **-**80°C for use. The amount of nucleic acid leakage was detected with supernatant at 260 nm (OD_260_), and the protein leakage was measured with a Bradford Protein Assay Kit (Nanjing Jiancheng Bioengineering Institute, China). Membrane lipid peroxidation was evaluated using MDA as an indicator (Ma et al., 2019). MDA content was determined by the MDA assay kit (Nanjing Jiancheng Bioengineering Institute, China). Each treatment was conducted in triplicate.

### 2.8 Transcriptomic analysis

To elucidate the global transcriptomic alterations induced by 2-HE treatment in *B. cinerea*, mycelia exposed to 2-HE (0.05 µL cm^-3^) and control groups treated with an equivalent volume of sterile distilled water were submitted to Megge Biotechnology (Shanghai, China) for transcriptome analysis. Transcriptome sequencing was performed using the Illumina sequencing platform, and differential expression genes (DEGs) were analyzed using the DESeq2 R package (screening criteria: p_adj_ ≤ 0.05). All identified DEGs were further analyzed by gene ontology (GO) and Kyoto Encyclopedia of Genes (KEGG) enrichment.

### 2.9 qPCR

To validate the RNA-seq results, six differentially expressed genes (DEGs) were chosen for qPCR verification. Mycelia total RNA was isolated using the Fungal RNA Kit (Omega, USA). qPCR amplification employed the RR820A and RR047A reagents from Baori Medical Technology Co., Ltd. (Beijing, China). Reference genes were selected based on Hua et al. (2019), and additional primer sequences were designed via Primer Premier 6 software (Premier, Canada), detailed in **Supplementary Table S1**. *Bcactin* served as the internal reference gene, and relative gene expression levels were calculated using the 2^-ΔΔCt^ method. The qPCR operational parameters are provided in **Supplementary Table S2**. Each experimental condition was replicated three times to ensure consistency.

### 2.10 Statistical analysis

The experimental data in the manuscript were analyzed using SPSS software (version 25.0) (IBM, New York, United States). Each result was represented by means ± standard error (SE), followed by variance analysis using Duncan’s multiple range test and T-test, and a significant difference (P < 0.05) was indicated with different lowercase letters.

## 3 Results

### 3.1 Effect of 2-HE on *B. cinerea* mycelial growth

**Figure 1:**
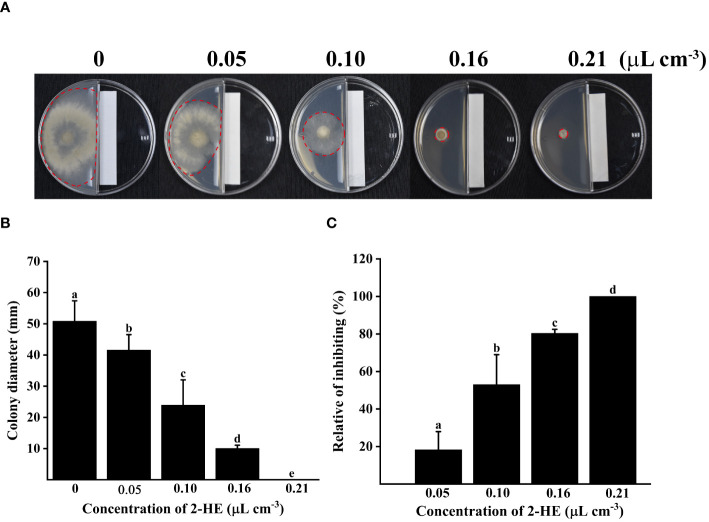
Varying concentrations of 2-HE (0, 0.05, 0.10, 0.16, and 0.21 μL cm^-3^) inhibited *B*. *cinerea*, in which 0 concentration was treated with an equal volume of distilled water instead of the maximum dose of 2-HE treatment (0.21 μL cm^-3^ H_2_O) as the control group. **(A)** Effect of 2-HE on the colony morphology of *B*. *cinerea*; **(B)** Colony diameter; **(C)** Relative of inhibiting. Duncan’s test was adopted, with different lowercase letters indicating significant differences (P<0.05), and the error bar in the figure represents the standard error.

2-HE significantly inhibited *B. cinerea* mycelial growth in a dose-dependent manner (**Figure 1**). At a concentration of 2-HE of 0.05 µL cm^-3^, the inhibition rate of growth was recorded at 18.35%. Notably, mycelial expansion was entirely halted at the 0.21 µL cm^-3^ 2-HE treatment, at which point the relative inhibition rate escalated to 100%. These findings demonstrate the potent inhibitory effect of 2-HE on *B. cinerea* mycelial growth.

### 3.2 Antifungal efficacy on harvested tomatoes

**Figure 2:**
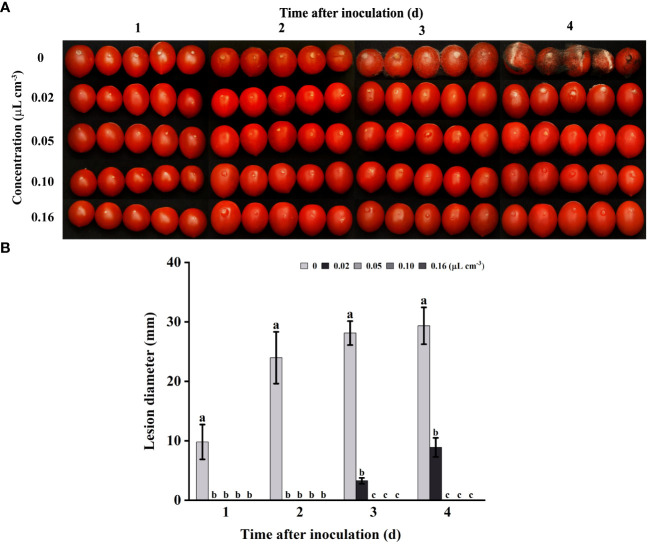
2-HE diminished the incidence of gray mold on tomatoes. **(A)** Tomatoes inoculated with *B*. *cinerea* and subsequently treated with varying concentrations of 2-HE (0, 0.02, 0.05, 0.10, and 0.16 μL cm^-3^, with the 0 µL cm^-3^ serving as the control); **(B)** Lesion diameter. Duncan’s test was adopted, with different lowercase letters indicating significant differences (P<0.05) and the error bar in the figure represents the standard error.

In this study, tomatoes were chosen as the subject to investigate the antifungal properties of 2-HE against gray mold in tomatoes. As shown in **Figure 2**, with increasing doses of 2-HE, the antifungal efficacy of 2-HE on tomatoes was significant *in vivo*, completely inhibiting gray mold at a dosage of 0.05 μL cm^-3^. In addition, lesions on tomatoes treated with 0.02 µL cm^-3^ of 2-HE were approximately 69.69% smaller in diameter than those on the control group by the fourth day. These results indicate that 2-HE possesses antifungal activity on postharvest tomatoes.

### 3.3 Effect of 2-HE on the antioxidant enzyme activity in tomatoes

**Figure 3:**
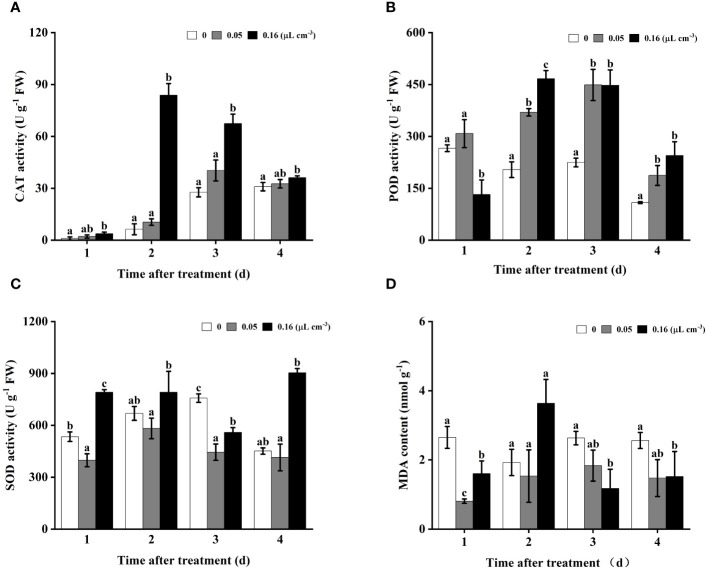
The antioxidant activities (SOD, POD, and CAT) and MDA content in tomatoes infected with *B*. *cinerea* were investigated at 2-HE concentrations of 0 (control), 0.05, and 0.16 μL cm^-3^. **(A)** CAT, **(B)** POD, **(C)** SOD, and **(D)** MDA content. Duncan’s test was adopted, with different lowercase letters indicating significant differences (P<0.05) and the error bar in the figure represents the standard error.

As shown in **Figure 3A**, throughout the entire storage period monitored, catalase (CAT) activity in the tomato fruit treated with 0.16 µL cm^-3^ of 2-HE was significantly higher than that in the control group. In contrast, at a 2-HE concentration of 0.05 µL cm^-3^, the difference in CAT activity between the treated and control groups was not statistically significant. These findings suggest a concentration-dependent relationship between 2-HE and CAT activity activation in tomato fruits. In addition, peroxidase (POD) activity in samples treated with 2-HE at both 0.05 and 0.16 µL cm^-3^ was significantly higher than the control. Interestingly, on the first day, POD activity was significantly reduced in the 0.16 µL cm^-3^ 2-HE treatment compared to the control (**Figure 3B**). With regard to superoxide dismutase (SOD) activity, treatment with 0.16 µL cm^-3^ of 2-HE resulted in significantly higher levels than the control at 1, 2, and 4 days; however, an unexpected decrease below control levels was observed at 2 days (**Figure 3C**).

Malondialdehyde (MDA) content is a widely recognized marker of lipid peroxidation in cell membranes. The study revealed that at a 2-HE concentration of 0.05 µL cm^-3^, the MDA levels were significantly reduced compared to the control on the first day of the storage period; however, no significant differences were observed at 2–4 days. Conversely, with 2-HE at 0.16 µL cm^-3^, MDA levels were substantially lower than those of the control at 1, 3, and 4 days (**Figure 3D**).

### 3.4 Effects of 2-HE on tomato quality

**Figure 4:**
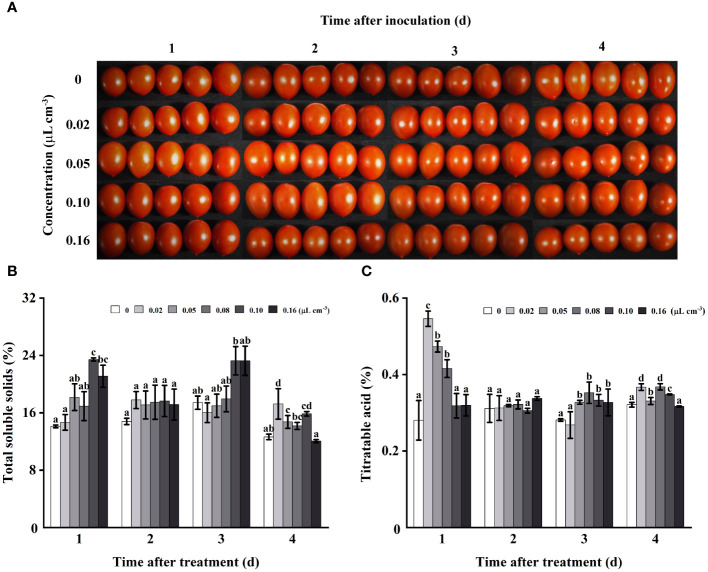
The appearance and contents of TSS and TA in healthy tomato fruits fumigated with varying 2-HE concentrations (0, 0.02, 0.05, 0.10, and 0.16 μL cm^-3^; this 0 μL cm^-3^ treatment used an equal volume of distilled water as the control group) were evaluated over a storage period of 1, 2, 3, and 4 days. **(A)** Effects of 2-HE on the tomato appearance; **(B)** TSS and **(C)** TA content in tomato. Duncan’s test was adopted, with different lowercase letters indicating significant differences (P < 0.05) and the error bar in the figure represents the standard error.

As storage duration increased, no significant impact of 2-HE on tomato appearance was observed (**Figure 4A**). The influence of 2-HE on total soluble solids (TSS) content in healthy tomatoes was also evaluated. It was determined that 2-HE concentrations of 0.02, 0.05, and 0.08 µL cm^-3^ did not significantly affect TSS content at 1, 2, or 3 days of storage. Notably, during a 2-day storage period, varying 2-HE concentrations did not significantly alter the TSS, thereby effectively maintaining the TSS content in the tomato fruits (**Figure 4B**). Regarding titratable acid (TA), the content in tomatoes varied across different storage durations and 2-HE concentrations. On the first day, TA content increased significantly when treated with 2-HE at 0.02, 0.05, and 0.08 µL cm^-3^. Similar to TSS, TA levels did not change significantly on the second day and were not influenced by 2-HE concentration (**Figure 4C**).

### 3.5 Effects of 2-HE on spore activity and membrane integrity of *B. cinerea*

**Figure 5:**
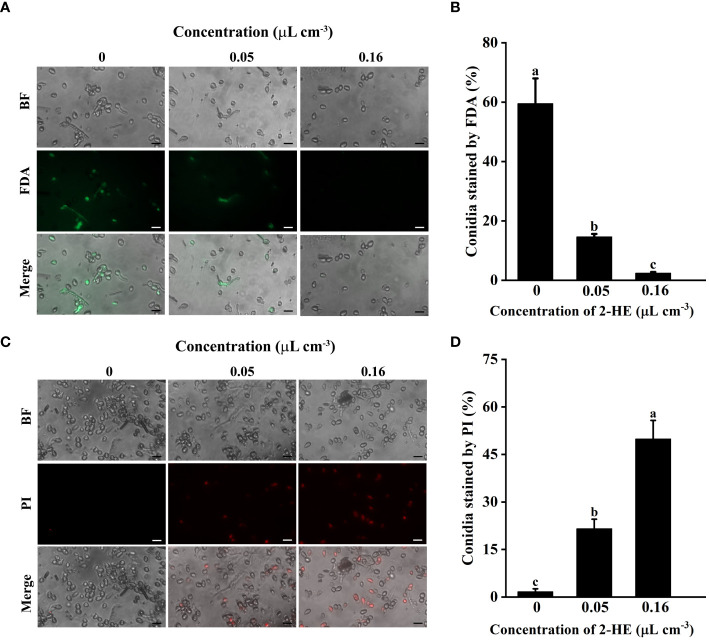
This study assessed conidial viability and membrane integrity following 2-HE treatment. **(A)** Effect of 2-HE treatment on the conidial activity of *B*. *cinerea*. Bar = 20 µm; **(B)** Conidia stained by FDA; **(C)** Effect of 2-HE treatment on the conidia membrane integrity of *B*. *cinerea*. Bar = 20 µm; **(D)** Conidia stained by PI. Duncan’s test was adopted, with different lowercase letters indicating significant differences (P<0.05) and the error bar in the figure represents the standard error.

The proportion of conidia stained with fluorescein diacetate (FDA) declined as the 2-HE concentration increased. At a 2-HE concentration of 0.16 µL cm^-3^, only 2.45% of the conidia were stained with FDA, suggesting a significant loss of viability (**Figures 5A, B**). Conversely, the proportion of conidia stained with propidium iodide (PI) increased inversely with the FDA results. With the 2-HE concentration at 0.16 µL cm^-3^, 49.9% of conidia were stained with PI, indicative of most conidia membrane disruption (**Figures 5C, D**). These findings demonstrate that 2-HE treatment reduces conidial viability and compromises membrane integrity.

### 3.6 Effect of 2-HE on cytoplasmic leakage and membrane lipid peroxidation

**Figure 6:**
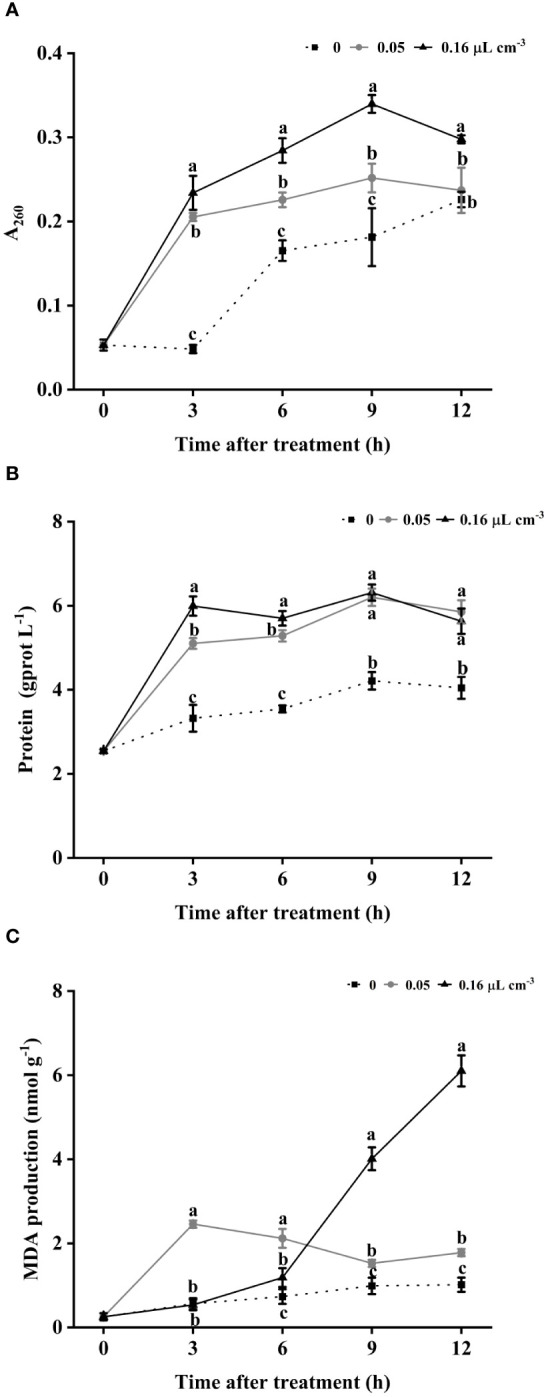
2-HE-induced cytoplasmic leakage and lipid peroxidation. **(A)** Nucleic acids; **(B)** soluble proteins; and **(C)** MDA content. Duncan’s test was adopted, with different lowercase letters indicating significant differences (P<0.05) and the error bar in the figure represents the standard error.

Given the high proportion of conidia stained by PI, we investigated nucleic acid and protein leakage as well as membrane lipid peroxidation in *B. cinerea* mycelia. As shown in **Figure 6A**, the control group exhibited lower levels of nucleic acid leakage than the 2-HE-treated group throughout the observation period. Comparatively, the content of nucleic acid leakage was positively correlated with the concentration of 2-HE; specifically, the group treated with 0.05 µL cm^-3^ had significantly less leakage than the 0.16 µL cm^-3^ group. Protein leakage was consistently higher in all 2-HE-treated groups than in the control group and peaked at 9 h of treatment (**Figure 6B**). Similarly, MDA levels in 2-HE-treated *B. cinerea* were significantly elevated compared to the control across all time points (**Figure 6C**). These findings suggest that 2-HE compromises the membrane integrity of *B. cinerea*, causing cytoplasmic leakage and membrane lipid peroxidation.

### 3.7 RNA-Seq analysis of *B. cinerea* under 2-HE stress

**Figure 7:**
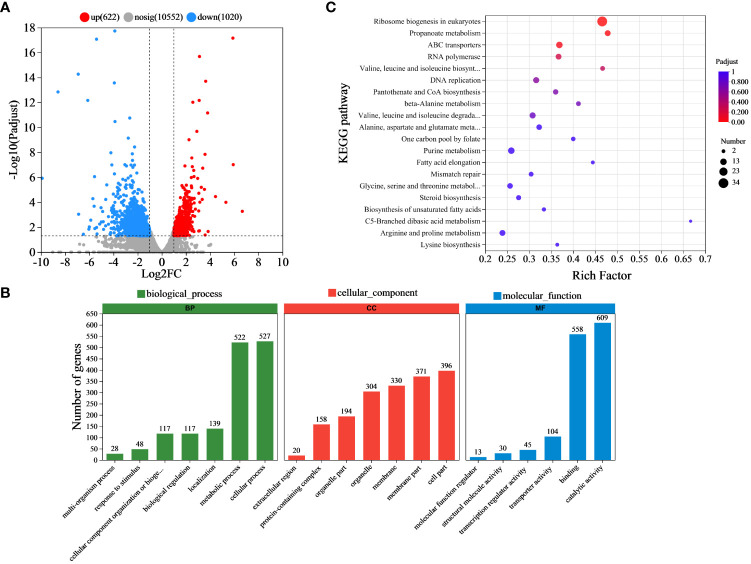
DEGs analysis. **(A)** Differential gene expression was analyzed by a volcano map, including 622 upregulated genes and 1,020 downregulated genes. **(B)** GO function classification statistics. **(C)** KEGG enrichment pathway.

To elucidate the molecular mechanism of 2-HE antifungal activity, the transcriptome method was used to analyze the whole gene of *B. cinerea* under 2-HE stress. Following treatment with 0.05 μL cm^-3^ of 2-HE, various RNA data for *B. cinerea* were obtained by RNA-Seq analysis. Compared to the control, 1,642 transcripts (622 upregulated and 1,020 downregulated) exhibited significant differential expression in the 2-HE-treated group (**Figure 7A**), with the top 20 DEGs shown in **Supplementary Table S3**. Through GO analysis, it was revealed that these DEGs were predominantly associated with biological processes, including metabolic and cellular processes. Within the cellular component category, the DEGs were related to cell part, membrane part, membrane, and organelle. In terms of molecular function, a significant proportion of the DEGs were involved in catalytic activity, binding, and transporter activity (**Figure 7B**). These findings indicate that the DEGs of *B. cinerea* treated with 2-HE primarily regulate cellular processes, metabolic processes, cell parts, membrane parts, membranes, organelles, catalytic activity, binding, and transporter activity.

The KEGG pathway enrichment analysis was performed to categorize the DEGs and further investigate the molecular mechanisms underlying the antifungal activity of 2-HE against *B. cinerea* (**Figure 7C**). The analysis identified the top 20 significantly enriched pathways (**Supplementary Table S4**), which predominantly included ribosome biogenesis in eukaryotes (map03008), propanoate metabolism (map00640), ABC transporters (map02010), and RNA polymerase (map03020). Additionally, several amino acid-related metabolic pathways were enriched, notably those involved in the biosynthesis and degradation of valine; leucine and isoleucine biosynthesis (map00290); valine, leucine, and isoleucine degradation (map00280); beta-alanine metabolism (map00410); alanine, aspartate, and glutamate metabolism (map00250); lysine biosynthesis (map00300); glycine, serine, and threonine metabolism (map00260); and arginine and proline metabolism (map00330). Validation of the relative expression levels of selected genes corroborated the transcriptomic findings, as indicated by a high correlation coefficient of 0.96668, thus confirming the reliability of the transcriptome data (**Supplementary Figure S1**).

### 3.8 DEGs related to amino acid metabolism and ABC transporters

**Figure 8:**
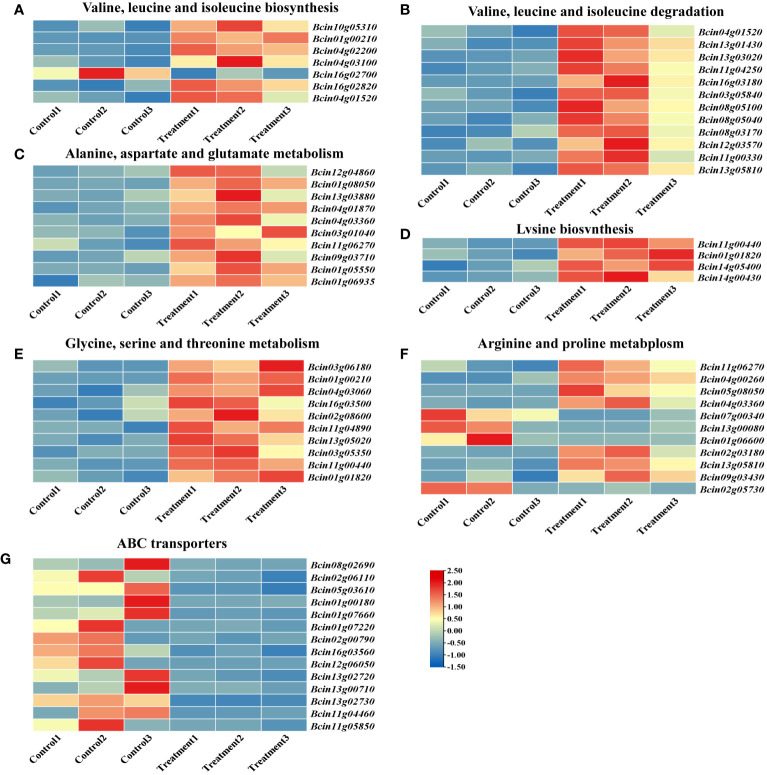
Heatmap of the gene expression for KEGG pathway-enriched amino acid metabolism. **(A)** valine, leucine, and isoleucine biosynthesis; **(B)** valine, leucine, and isoleucine degradation; **(C)** alanine, aspartate, and glutamate metabolism; **(D)** lysine biosynthesis; **(E)** glycine, serine, and threonine metabolism; **(F)** arginine and proline metabolism; and **(G)** ABC transporters.

Analysis of the top 20 enriched metabolic pathways via KEGG revealed that six pathways were associated with amino acid metabolism. To further elucidate the molecular mechanism of 2-HE in *B. cinerea*, TBtools was employed to construct a heatmap of differential gene expression across these six amino acid metabolism pathways. The valine, leucine, and isoleucine biosynthesis pathway (map00290) exhibited enrichment for seven DEGs, six of which (*Bcin10g05310*, *Bcin01g00210*, *Bcin04g02200*, *Bcin04g03100*, *Bcin16g02820*, and *Bcin04g01520*) were upregulated, suggesting an acceleration of these biosynthetic processes by 2-HE treatment (**Figure 8A**; **Supplementary Table S5**). Concomitantly, the pathway for degradation of valine, leucine, and isoleucine (map00280) was also activated, as evidenced by 12 upregulated DEGs (**Figure 8B**; **Supplementary Table S6**). Similarly, the pathways for alanine, aspartate, and glutamate metabolism (map00250); lysine biosynthesis (map00300); and glycine, serine, and threonine metabolism (map00260) were enriched with 10, 4, and 10 upregulated DEGs, respectively (**Figures 8C–E**; **Supplementary Tables S7**-**S10**). Under 2-HE treatment, 11 DEGs contributing to arginine and proline metabolism (map00330) were identified, with seven (*Bcin11g06270*, *Bcin04g00260*, *Bcin05g08050*, *Bcin04g03360*, *Bcin02g03180*, *Bcin13g05810*, *Bcin09g03430*) showing significant upregulation and four (*Bcin07g00340*, *Bcin13g00080*, *Bcin01g06600*, *Bcin02g05730*) being significantly downregulated (**Figure 8F**; **Supplementary Table S10**). In summary, these findings indicate that 2-HE treatment enhances amino acid metabolism in *B. cinerea*.

KEGG pathway enrichment analysis indicated that 2-HE treatment also affected the membrane transport system of *B. cinerea*. ABC transporters, which utilize the energy derived from ATP hydrolysis to facilitate the uptake of nutrients such as amino acids and sugars, are instrumental in promoting cell growth (Locher, 2016). Heatmap analysis showed that in cells treated with 2-HE, 14 DEGs corresponded to ABC transporters, and critically, all of these DEGs were significantly downregulated (**Figure 8G**). This evidence suggests that 2-HE treatment impedes the membrane transport system of *B. cinerea*.

### 3.9 2-HE inhibited ABC transporters in *B. cinerea*

The heatmap presented in **Figure 8G** reveals that 2-HE treatment leads to the downregulation of genes associated with ABC transporters. As shown in **Table 2**, *Bcin01g00180*, *Bcin12g06050*, and *Bcin13g02730* exhibit markedly higher downregulation, at 3.80, 3.72, and 3.89 times, respectively. Notably, genes implicated in *B. cinerea* multidrug resistance, such as *BcatrA* (*Bcin11g04460*), *BcatrB* (*Bcin13g00710*), and *BcatrD* (*Bcin13g02720*), also trended downward, with respective fold changes of 2.43, 2.71, and 1.16.

**Table 2:** 2-HE-downregulated ABC-related genes.

Gene _ id	Gene name	Log2FoldChange	p-value
*Bcin08g02690*	–	-2.102	0.000
*Bcin02g06110*	–	-1.757	0.007
*Bcin05g03610*	–	-1.318	0.000
*Bcin01g00180*	–	-3.808	0.000
*Bcin01g07660*	–	-2.315	0.000
*Bcin01g07220*	–	-1.727	0.006
*Bcin02g00790*	–	-2.028	0.001
*Bcin16g03560*	–	-1.854	0.000
*Bcin12g06050*	–	-3.723	0.000
*Bcin13g02720*	*BcatrD*	-1.167	0.002
*Bcin13g00710*	*BcatrB*	-2.715	0.000
*Bcin13g02730*	*-*	-3.893	0.000
*Bcin11g04460*	*BcatrA*	-2.431	0.000
*Bcin11g05850*	–	-1.773	0.001

## 4 Discussion

Biogenic secondary metabolites offer a sustainable alternative to synthetic pesticides for controlling plant diseases (Valotteau et al., 2017; Chen et al., 2021). 2-HE, a VOC, presents a less toxic, safer, and more reliable option than traditional fungicides for managing diseases in tomatoes (Gong et al., 2022). For instance, Du et al. (2022) observed that 2-HE effectively reduced the incidence of Fusarium crown and root rot in tomatoes. These findings suggest that 2-HE has great potential to inhibit tomato disease.

Mycelium growth represents a critical way through which *B. cinerea* infects hosts, and targeting this process is a primary strategy for preventing grey mold (Bi et al., 2023; Liu K. et al., 2023). Song et al. (2024) reported that 2-nonanol, produced from *Bacillus aryabhattai*, markedly suppresses *Penicillium expansum* mycelia growth. *In vivo* studies demonstrate that biogenic secondary metabolites reduce postharvest diseases in fruits and vegetables; for example, reveromycin E, isolated from *Streptomyces*, shows substantial inhibition of tomato *B. cinerea* and *Penicillium italicum* (Nguyen et al., 2021). In addition, 3-methylbutan-1-ol, produced by endophytic bacteria, significantly reduces the incidence of tomato gray mold (Chaouachi et al., 2021). Investigations have confirmed that such metabolites curtail the mycelial growth of pathogenic fungi both *in vivo* and *in vitro* and exhibit a dose–response relationship, with an increase in metabolite concentration leading to heightened fungal suppression. Notable compounds displaying this effect include epsilon-polylysine (Li et al., 2019), L-cysteine (Wang et al., 2023), and β-glucan (Zhao et al., 2020). This study demonstrates that 2-HE substantially curtails *B. cinerea* mycelial growth under both *in vivo* and *in vitro* conditions in a dose-dependent manner. These findings suggest that 2-HE has the potential to manage *B. cinerea* infection by hindering mycelium growth.

The conidium causes the long-distance transmission of gray mold, so the inactivation of conidia is also one of the ways to prevent gray mold (Dai et al., 2021). Studies have shown that the use of biological bacteria and their secondary metabolites also reduces the conidial activity of pathogenic fungi (Russi et al., 2024), for example, hinokitiol (Wang et al., 2020) and magnolol (Cui et al., 2021). In this study, we also discovered that 2-HE markedly reduces the conidial activity of *B. cinerea*. This suggests that 2-HE reduces the possibility of long-distance transmission of *B. cinerea* by inhibiting spore activity.

Inducing host resistance is a central strategy for controlling postharvest fruit and vegetable diseases (Kesel et al., 2021; Prusky and Romanazzi, 2023). Augmenting the activity of defense-related enzymes constitutes a significant aspect of this resistance against pathogenic fungi (Raynaldo et al., 2021). POD, CAT, and SOD are the pivotal antioxidant enzymes in fruits and vegetables; their heightened activity signals an enhanced capacity for reactive oxygen species detoxification (Jing et al., 2020; Dou et al., 2021). Relevant studies indicate that the application of bioderived secondary metabolites activates these enzymes activities, thereby fortifying host resistance to fungal pathogens (Wu et al., 2023; Xing et al., 2023). Our study demonstrated that the activities of CAT, SOD, and POD were related to the concentration of 2-HE. Notably, compared to the control, 2-HE activated the antioxidant enzyme activity in the tomato host, substantially mitigating the invasion by gray mold. Malondialdehyde (MDA), a primary product of membrane lipid peroxidation, is commonly used to gauge oxidative damage (Ling et al., 2023). Throughout storage, MDA content at 0.05 μL cm^-3^ of 2-HE was lower than that in the control group, whereas at 0.16 μL cm^-3^, MDA content surpassed the control on the second day of storage. The results indicated that 2-HE diminishes tomato cell membrane peroxidation at low concentrations (0.05 μL cm^-3^), identifying its potential as a preservative. In general, 2-HE acts as an antifungal agent by reducing membrane peroxidation damage in tomato fruits, activating the defense response, inducing resistance, and ultimately suppressing the incidence of tomato gray mold at an optimal concentration.

In managing postharvest decay, preserving the appearance, flavor, texture, and nutritional value of fruits and vegetables is of paramount importance (Chen et al., 2021; Hosseini et al., 2023). Wang et al. (2024) demonstrated that natamycin does not adversely impact the quality attributes of fruits, such as grape stem appearance, berry hardness, total soluble solids (TSS), and titratable acidity (TA). Similar results were also reported in thymol (Ding et al., 2023), dimethyl disulfide, and dimethyl trisulfide (Wang et al., 2021). Additionally, 2-ethyl-5-methylpyrazine was found to enhance mango postharvest quality by increasing TSS, total phenol, total proline, total carotenoid, total flavonoid, and fruit firmness (Archana et al., 2021). Our study indicates that 2-HE neither compromises the external appearance of tomato fruit nor diminishes its postharvest quality.

The plasma membrane plays a pivotal role in sustaining normal cellular function in fungal pathogens, rendering it a frequent target for antifungal agents (Liu R. et al., 2023; Wei et al., 2021). Numerous studies demonstrate that various antifungal compounds compromise membrane integrity, result in the leakage of cytoplasmic contents, and lead to spore mortality (Ma et al., 2019; Zhang M. et al., 2020). Our findings reveal that 2-HE treatment notably reduces spore activity and impairs membrane integrity. Moreover, elevated levels of soluble carbohydrates, nucleic acids, and MDA were detected in *B. cinerea* subjected to 2-HE treatment. These outcomes suggest that 2-HE disrupts membrane permeability, triggers cytoplasmic leakage, and induces membrane lipid peroxidation, findings that align with those from studies on 3-Octanol (Zhang et al., 2023), 2-Phenylethanol (Zou et al., 2022), and 2,3-Butanedione (Li et al., 2022).

ABC transporters facilitate the translocation of not only a wide array of nutrients but also exogenous drugs, with certain transporters playing a pivotal role in cell growth and antifungal resistance (Viglas and Olejnikova, 2021). To date, 14 ABC transporters (*BcatrA* to *BcatrN*) have been characterized, and of these, the *BcatrB*, *BcatrD*, and *BcatrK* genes are implicated in the multidrug resistance of *B. cinerea*, primarily functioning in the expulsion of toxic fungi compounds (Stefanato et al., 2009; Wu et al., 2024). Research has revealed that antifungal agents impinge on the functionality of pathogen ABC transporters by modulating ABC gene expression (Wu et al., 2024). Our investigations indicate that *BcatrB* and *BcatrD* genes were significantly downregulated following 2-HE treatment by 2.71 and 1.16 times, respectively, suggesting impaired *B. cinerea* ability to extrude 2-HE as a toxic substance. Moreover, other genes associated with ABC transporters were also substantially downregulated. Such findings resonate with observations of certain antifungal substances, including resveratrol (Vermeulen et al., 2001), psoralen, and eugenol (Hayashi et al., 2002). These RNA-Seq data demonstrate that 2-HE impedes the ABC transporters pathway, corroborating the notion that 2-HE compromises the membrane structure of *B. cinerea* and consequently disrupts membranal transport functionality.

Amino acids play a crucial role in promoting cell growth and survival by serving as vital nutrients (Li and Zhang, 2023). Disrupted amino acid metabolism often results in cell death (Jin et al., 2020). Our research found that most DEGs related to amino acid metabolism were upregulated, suggesting that 2-HE contributes to nutrient depletion in *B. cinerea* cells. Consequently, treatment with 2-HE not only obstructs the membrane transport pathway but also hastens intracellular amino acid metabolism within *B. cinerea* cells. This destruction of the membrane structure, along with the inhibition of the membrane transport pathway and the acceleration of intracellular amino acid metabolism, culminates in the cells’ inability to assimilate nutrients, leading to eventual nutrient depletion.

## 5 Conclusion

In summary, 2-HE exhibits a potent antifungal effect in both *in vitro* and *in vivo* conditions, enhancing the activity of defense enzymes in tomatoes. At the same time, 2-HE does not adversely affect the appearance or quality of tomatoes. The antifungal mechanism of 2-HE against *B. cinerea* involves the acceleration of intracellular amino acid metabolism, disruption of membrane structure, and inhibition of the membrane transport system, resulting in the depletion of intracellular nutrients and the eventual demise of *B. cinerea*. Consequently, 2-HE represents a promising natural agent for the control of postharvest gray mold in tomatoes.

## Data availability statement

The data presented in study are deposited in the NCBI repository, accession number PRJNA114723.

## Author contributions

FW: Writing – review & editing, Writing – original draft, Methodology, Conceptualization. HW: Writing – original draft, Data curation. YL: Writing – review & editing, Visualization, Data curation. ZQ: Writing – original draft, Visualization. BZ: Writing – original draft, Software, Writing – review & editing. SF: Conceptualization, Writing – review & editing, Supervision, Funding acquisition. XL: Writing – review & editing, Supervision, Funding acquisition, Data curation.
