# Supplementary material for: 2-Heptanol inhibits Botrytis cinerea by accelerating amino acid metabolism and retarding membrane transport
Source: Front Plant Sci. 2024 Jun 3;15:1400164. doi: 10.3389/fpls.2024.1400164 (PMC11180792; doi:10.3389/fpls.2024.1400164)
Supplement: Supplementary file 1 [file DataSheet_1.docx]

**Supporting Information**

**Table S1.** Gene and primers used in RT-qPCR validation

| Gene ID | Gene name | Gene description | F (5’-3’) |
| --- | --- | --- | --- |
| *Bcin12g06350* | *Bchex6* | cell growth and death | TGGTTGGTGCTGCTGAGATGT |
|  |  |  | AGTTGGATGCGGTAGCGAGAG |
| *Bcin08g06670* | *Bchop1* | cell growth and death | ACACAACCGATACCCGCCTAC |
|  |  |  | ACTGCTTCCGACGCTTCCTC |
| *Bcin03g00150* | *Bcdoc1* | cell growth and death | CACGATTCAACCACAGGCACAG |
|  |  |  | GCTTCAACTGCGACATCTTCCA |
| *Bcin08g05260* | *Bcmdm10* | cell growth and death | TTGCCTCTCCGTACCACTTCG |
|  |  |  | AACAGCCGTAATCCTCACTTGC |
| *Bcin14g01870* | *Bchhk5* | mitophagy | TTGTGCTCGCTCTCGCTACC |
|  |  |  | TCTCTTGCTTCGTTCCACTCGG |
| *Bcin05g02500* | *Bcactin* | housekeeping genes | CTCTATTCAAGCCGTCCTCTCC |
|  |  |  | TAATCAGTCAAATCACGACCAGC |

**Table S2:** qPCR operation parametric

| Process | Steps | Temperature (℃) | Time (m: s) | Cycle |
| --- | --- | --- | --- | --- |
| Initial denaturation | 1 | 95.0 | 10:00 | 1 |
| Quantitative analysis | 2 | 95.0 | 00:05 | 40 |
|  | 3 | 60.0 | 00:30 |  |
|  | 4 | 72.0 | 00:30 |  |
| melting curve | 5 | melt | 00:15 | 1 |

**Table S3.** Differential gene GO enrichment entries.

| GO-enrich entries | Type | Gene ID |
| --- | --- | --- |
| maturation of SSU-rRNA from tricistronic rRNA transcript (SSU-rRNA, 5.8S rRNA, LSU-rRNA  (GO:0000462)  (BP) | up | *Bcin15g02620, Bcin09g02190, Bcin06g06610, Bcin15g01260, Bcin14g02020, Bcin01g09800,*  *Bcin03g06050, Bcin02g08110, Bcin09g02180, Bcin11g03140, Bcin09g02170, Bcin06g06550* |
| rRNA processing  (GO:0006364)  (BP) | up | *Bcin04g03020, Bcin15g01550, Bcin02g04190, Bcin15g02620, Bcin02g06650, Bcin09g02190,*  *Bcin02g08540, Bcin14g00960, Bcin14g04850, Bcin11g02220, Bcin06g06610, Bcin02g04540,*  *Bcin06g03200, Bcin07g02450, Bcin10g04050, Bcin07g03950, Bcin02g04160, Bcin06g03290,*  *Bcin09g01390, Bcin03g02950, Bcin15g01260, Bcin08g01510, Bcin03g07200, Bcin03g05940,*  *Bcin14g02020, Bcin01g09800, Bcin05g04660, Bcin16g02330, Bcin07g01580, Bcin07g05110,*  *Bcin11g02250, Bcin05g02880, Bcin05g05790, Bcin07g05070, Bcin06g06940, Bcin15g03900,*  *Bcin03g06050, Bcin02g08110, Bcin12g01820, Bcin05g06800, Bcin03g05220, Bcin01g07420,*  *Bcin10g03950, Bcin12g01810, Bcin12g02850, Bcin06g06690, Bcin04g03860, Bcin04g01690,*  *Bcin08g04150, Bcin08g04270, Bcin05g07440, Bcin04g03400, Bcin01g04710, Bcin12g05410,*  *Bcin11g03130, Bcin06g00260, Bcin11g02350, Bcin14g02520, Bcin05g00070, Bcin10g04550,*  *Bcin03g06280, Bcin09g02180, Bcin11g03140, Bcin09g02170, Bcin09g04660, Bcin06g00730,*  *Bcin06g06550, Bcin06g06810, Bcin03g06530, Bcin01g05400, Bcin04g05800* |
|  | down | *Bcin12g06650* |
| endonucleolytic cleavage involved in rRNA processing  (GO:0000478) (BP) | up | *Bcin15g02620, Bcin09g02190, Bcin06g06610, Bcin07g02450, Bcin02g04160, Bcin01g09800,*  *Bcin05g04660, Bcin05g05790, Bcin07g05070,*  *Bcin04g01690, Bcin05g00070, Bcin03g06280,*  *Bcin09g02180, Bcin06g00730, Bcin01g10480,*  *Bcin06g06550, Bcin06g06810, Bcin01g05400* |
| endonucleolytic cleavage of tricistronic rRNA transcript (SSU-rRNA, 5.8SrRNA, LSU-rRNA  (GO:0000479) (BP) | up | *Bcin15g02620, Bcin09g02190, Bcin06g06610,*  *Bcin07g02450, Bcin02g04160, Bcin01g09800,*  *Bcin05g04660, Bcin05g05790, Bcin07g05070,*  *Bcin04g01690, Bcin05g00070, Bcin03g06280,*  *Bcin09g02180, Bcin06g00730, Bcin01g10480,*  *Bcin06g06550, Bcin06g06810, Bcin01g05400* |
| maturation of SSU-rRNA  (GO:0030490) (BP) | up | *Bcin15g02620, Bcin09g02190, Bcin06g06610,*  *Bcin15g01260, Bcin14g02020, Bcin01g09800,*  *Bcin05g02880, Bcin06g06940, Bcin03g06050,*  *Bcin02g08110, Bcin05g06800, Bcin09g02180,*  *Bcin11g03140, Bcin09g02170, Bcin06g06550* |
| cleavage involved in rRNA processing  (GO:0000469) (BP) | up | *Bcin15g02620, Bcin09g02190, Bcin14g04850,*  *Bcin11g02220, Bcin06g06610, Bcin07g02450,*  *Bcin02g04160, Bcin15g01260, Bcin03g05940,*  *Bcin01g09800, Bcin05g04660, Bcin05g05790,*  *Bcin07g05070, Bcin04g01690, Bcin05g00070,*  *Bcin03g06280, Bcin09g02180, Bcin06g00730,*  *Bcin01g10480, Bcin06g06550, Bcin06g06810,*  *Bcin01g05400* |
| cellular component biogenesis  (GO:0044085) (BP) | up | *Bcin15g01550, Bcin02g04190, Bcin02g06650,*  *Bcin02g08540, Bcin12g03770, Bcin14g00960,*  *Bcin14g04850, Bcin10g04050, Bcin07g03950,*  *Bcin02g04160, Bcin01g03220, Bcin03g02950,*  *Bcin15g01260, Bcin03g07200, Bcin03g05940,*  *Bcin14g02020, Bcin01g09800, Bcin16g02330,*  *Bcin07g01580, Bcin07g05110, Bcin05g05790,*  *Bcin02g03570, Bcin07g05070, Bcin06g06940,*  *Bcin03g06050, Bcin12g01820, Bcin01g07420,*  *Bcin10g03950, Bcin03g00610, Bcin12g01810,*  *Bcin06g06690, Bcin05g04170, Bcin10g05890,*  *Bcin07g05660, Bcin08g04270, Bcin05g07440,*  *Bcin04g03400, Bcin01g04710, Bcin11g03130,*  *Bcin02g04460, Bcin14g02520, Bcin09g02180,*  *Bcin11g03140, Bcin09g04920, Bcin02g03580,*  *Bcin16g03140, Bcin08g00630, Bcin09g04660,*  *Bcin01g10480, Bcin06g06810, Bcin03g06530,*  *Bcin01g05400, Bcin08g05890, Bcin04g05800,*  *Bcin03g04160* |
| endonucleolytic cleavage to generate mature 5'-end of SSU-rRNA from (SSU-rRNA, 5.8SrRNA, LSU-rRNA)  (GO:0000472) (BP) | up | *Bcin15g02620, Bcin09g02190, Bcin06g06610,*  *Bcin07g02450, Bcin02g04160, Bcin01g09800,*  *Bcin05g04660, Bcin05g05790, Bcin05g00070,*  *Bcin03g06280, Bcin06g06550, Bcin06g06810,*  *Bcin01g05400* |
| ribonucleoprotein complex biogenesis  (GO:0022613) (BP) | up | *Bcin15g01550, Bcin02g04190, Bcin02g06650,*  *Bcin02g08540, Bcin12g03770, Bcin14g00960,*  *Bcin14g04850, Bcin07g03950, Bcin02g04160,*  *Bcin01g03220, Bcin03g02950, Bcin15g01260,*  *Bcin03g07200, Bcin03g05940, Bcin14g02020,*  *Bcin01g09800, Bcin16g02330, Bcin07g01580,*  *Bcin07g05110, Bcin05g05790, Bcin02g03570,*  *Bcin07g05070, Bcin06g06940, Bcin03g06050,*  *Bcin12g01820, Bcin01g07420, Bcin10g03950,*  *Bcin03g00610, Bcin12g01810, Bcin06g06690,*  *Bcin05g04170, Bcin10g05890, Bcin07g05660,*  *Bcin08g04270, Bcin05g07440, Bcin04g03400,*  *Bcin01g04710, Bcin11g03130, Bcin02g04460,*  *Bcin14g02520, Bcin09g02180, Bcin11g03140,*  *Bcin09g04920, Bcin02g03580, Bcin16g03140,*  *Bcin08g00630, Bcin09g04660, Bcin01g10480,*  *Bcin06g06810, Bcin03g06530, Bcin01g05400,*  *Bcin08g05890, Bcin04g05800, Bcin03g04160* |
| preribosome, large subunit precursor  (GO:0030687) (CC) | up | *Bcin02g04190, Bcin10g04050, Bcin03g02950,*  *Bcin16g02330, Bcin07g01580, Bcin07g05110,*  *Bcin11g02250, Bcin07g05070, Bcin06g02350,*  *Bcin06g06690, Bcin04g01690, Bcin05g04170* |
| Preribosome  (GO:0030684) (CC) | up | *Bcin02g04190, Bcin15g02620, Bcin09g02190,*  *Bcin02g08540, Bcin06g06610, Bcin07g02450,*  *Bcin10g04050, Bcin02g04160, Bcin01g03220,*  *Bcin03g02950, Bcin15g01260, Bcin14g02020,*  *Bcin01g09800, Bcin05g04660, Bcin16g02330,*  *Bcin07g01580, Bcin07g05110, Bcin11g02250,*  *Bcin05g02880, Bcin05g05790, Bcin15g03900,*  *Bcin06g02350, Bcin12g01820, Bcin03g05220,*  *Bcin06g06690, Bcin04g01690, Bcin05g04170,*  *Bcin10g05890, Bcin08g04270, Bcin05g07440,*  *Bcin01g04710, Bcin11g02350, Bcin14g02520,*  *Bcin05g00070, Bcin10g04550, Bcin09g02180,*  *Bcin09g02170, Bcin09g04660, Bcin06g00730,*  *Bcin01g10480, Bcin06g06550, Bcin06g06810,*  *Bcin01g05400, Bcin03g04160* |
| small-subunit processome  (GO:0032040)  (CC) | up | *Bcin15g02620, Bcin09g02190, Bcin02g08540,*  *Bcin06g06610, Bcin07g02450, Bcin02g04160,*  *Bcin01g03220, Bcin14g02020, Bcin01g09800,*  *Bcin05g02880, Bcin05g05790, Bcin12g01820,*  *Bcin03g05220, Bcin14g02520, Bcin05g00070,*  *Bcin10g04550, Bcin09g04660, Bcin06g06550,*  *Bcin06g06810* |
| RNA 5'-end processing  (GO:0000966) (BP) | up | *Bcin15g02620, Bcin09g02190, Bcin06g06610,*  *Bcin07g02450, Bcin02g04160, Bcin01g09800,*  *Bcin05g04660, Bcin05g05790, Bcin07g05070,*  *Bcin05g00070, Bcin03g06280, Bcin06g06550,*  *Bcin06g06810, Bcin01g05400* |
| maturation of LSU-rRNA  (GO:0000470)  (BP) | up | *Bcin15g01550, Bcin02g04190, Bcin14g04850,*  *Bcin07g03950, Bcin03g02950, Bcin14g02020,*  *Bcin16g02330, Bcin07g05110, Bcin11g02250,*  *Bcin06g06940, Bcin15g03900, Bcin06g06690,*  *Bcin08g04270, Bcin01g04710* |
| endonucleolytic cleavage in ITS1 to separate SSU-rRNA from 5.8S rRNA and LSU-rRNA from tricistronic rRNA transcript (SSU-rRNA, 5.8S rRNA, LSU-rRNA)  (GO:0000447) (BP) | up | *Bcin15g02620, Bcin09g02190, Bcin06g06610,*  *Bcin07g02450, Bcin02g04160, Bcin01g09800,*  *Bcin05g04660, Bcin05g05790, Bcin05g00070,*  *Bcin03g06280, Bcin06g00730, Bcin01g10480,*  *Bcin06g06550, Bcin06g06810, Bcin01g05400* |
| RNA phosphodiester bond hydrolysis  (GO:0090501)  (BP) | up | *Bcin15g02620, Bcin09g02190, Bcin01g03690,*  *Bcin14g04850, Bcin03g02290, Bcin11g02220,*  *Bcin06g06610, Bcin07g02450, Bcin02g04160,*  *Bcin02g02880, Bcin15g01260, Bcin03g05940,*  *Bcin03g07940, Bcin01g09800, Bcin05g04660,*  *Bcin05g05790, Bcin07g05070, Bcin02g03160,*  *Bcin04g01690, Bcin02g00630, Bcin05g00070,*  *Bcin03g06280, Bcin09g02180, Bcin06g00730,*  *Bcin01g10480, Bcin06g06550, Bcin06g06810,*  *Bcin01g05400* |
|  | down | *Bcin16g04490, Bcin12g06230* |
| RNA phosphodiester bond hydrolysis, endonucleolytic  (GO:0090502) (BP) | up | *Bcin15g02620, Bcin09g02190, Bcin06g06610,*  *Bcin07g02450, Bcin02g04160, Bcin02g02880,*  *Bcin15g01260, Bcin03g07940, Bcin01g09800,*  *Bcin05g04660, Bcin05g05790, Bcin07g05070,*  *Bcin02g03160, Bcin04g01690, Bcin02g00630,*  *Bcin05g00070, Bcin03g06280, Bcin09g02180,*  *Bcin06g00730, Bcin01g10480, Bcin06g06550,*  *Bcin06g06810, Bcin01g05400* |
|  | down | *Bcin16g04490, Bcin12g06230,* |
| endonucleolytic cleavage in 5'-ETS of tricistronic rRNA transcript (SSU-rRNA, 5.8S rRNA, LSU-rRNA)  (GO:0000480) (BP) | up | *Bcin15g02620, Bcin09g02190, Bcin06g06610,*  *Bcin07g02450, Bcin02g04160, Bcin01g09800,*  *Bcin05g04660, Bcin05g05790, Bcin05g00070,*  *Bcin03g06280, Bcin06g06550, Bcin06g06810,*  *Bcin01g05400* |
| rRNA 5'-end processing  (GO:0000967) (BP) | up | *Bcin15g02620, Bcin09g02190, Bcin06g06610,*  *Bcin07g02450, Bcin02g04160, Bcin01g09800,*  *Bcin05g04660, Bcin05g05790, Bcin05g00070,*  *Bcin03g06280, Bcin06g06550, Bcin06g06810,*  *Bcin01g05400* |
| ncRNA 5'-end processing  (GO:0034471) (BP) | up | *Bcin15g02620, Bcin09g02190, Bcin06g06610,*  *Bcin07g02450, Bcin02g04160, Bcin01g09800,*  *Bcin05g04660, Bcin05g05790, Bcin07g05070,*  *Bcin05g00070, Bcin03g06280, Bcin06g06550,*  *Bcin06g06810, Bcin01g05400* |

**Table S4.** Metabolic pathways after KEGG enrichment of DEGs

| **KEGG ID** | **Description** | **padj** | **Count** | **Gene ID** |
| --- | --- | --- | --- | --- |
| map03008 | Ribosome biogenesis in eukaryotes | 4.09E-9 | 34 | *Bcin14g02330, Bcin14g05230, Bcin01g01040, Bcin09g01390,*  *Bcin10g03950, Bcin05g02330,*  *Bcin07g02450, Bcin06g06610,*  *Bcin02g08110, Bcin15g02620,*  *Bcin04g03400, Bcin09g04660,*  *Bcin14g02520, Bcin10g04550,*  *Bcin02g08540, Bcin05g05790,*  *Bcin14g02020, Bcin16g03140,*  *Bcin14g04850, Bcin02g06650,*  *Bcin06g06810, Bcin03g05220,*  *Bcin05g07440, Bcin01g05400,*  *Bcin03g02290, Bcin07g01580,*  *Bcin06g02350, Bcin11g02250,*  *Bcin16g04670, Bcin15g01260,*  *Bcin05g06040, Bcin03g06050,*  *Bcin12g03160* |
| mp00640 | Propanoate metabolism | 0.007583896 | 11 | *Bcin07g06960, Bcin12g03570,*  *Bcin08g03170, Bcin03g05840,*  *Bcin14g02030, Bcin13g01430, Bcin13g03020, Bcin11g04250, Bcin09g01930, Bcin07g03110, Bcin04g03150* |
| mp02010 | ABC transporters | 0.018805050 | 14 | *Bcin08g02690, Bcin02g06110,*  *Bcin05g03610, Bcin01g00180,*  *Bcin01g07660, Bcin01g07220,*  *Bcin02g00790, Bcin16g03560,*  *Bcin12g06050, Bcin13g02720,*  *Bcin13g00710, Bcin13g02730,*  *Bcin11g04460, Bcin11g05850* |
| map03020 | RNA polymerase | 0.058270920 | 13 | *Bcin03g02430, Bcin06g05640,*  *Bcin02g02530, Bcin08g06090,*  *Bcin12g03590, Bcin05g05570,*  *Bcin12g02760, Bcin03g07720,*  *Bcin03g02430, Bcin13g04380,*  *Bcin06g05640, Bcin14g02770,*  *Bcin03g05790* |
| map00290 | Valine, leucine and isoleucine biosynthesis | 0.062846389 | 7 | *Bcin10g05310, Bcin01g00210,*  *Bcin04g02200, Bcin04g03100,*  *Bcin16g02700, Bcin16g02820, Bcin04g01520* |
| map0028 | Valine, leucine and isoleucine degradation | 0.085978544 | 12 | *Bcin04g01520, Bcin13g01430,*  *Bcin03g05840, Bcin08g05100,*  *Bcin08g05040, Bcin11g00330,*  *Bcin13g03020, Bcin16g03180,*  *Bcin08g03170, Bcin12g03570,*  *Bcin11g04250, Bcin11g04250* |
| map00410 | beta-Alanine metabolism | 0.091251825 | 7 | *Bcin03g01040, Bcin13g05810,*  *Bcin12g03030, Bcin12g03570,*  *Bcin08g03170, Bcin03g05840,*  *Bcin14g02030* |
| map00770 | Pantothenate and CoA biosynthesis | 0.096137721 | 9 | *Bcin04g02200, Bcin04g03100,*  *Bcin16g02700, Bcin16g02820,*  *Bcin04g01520, Bcin01g01850,*  *Bcin03g05920, Bcin12g03030,*  *Bcin13g05810* |
| map03030 | DNA replication | 0.102442242 | 12 | *Bcin08g00460, Bcin12g01680,*  *Bcin11g03470, Bcin14g00470,*  *Bcin11g05760, Bcin12g06740,*  *Bcin01g10610, Bcin12g01760,*  *Bcin03g04190, Bcin14g01800,*  *Bcin02g00920, Bcin02g02880* |
| map00250 | Alanine, aspartate and glutamate metabolism | 0.10345174 | 10 | *Bcin12g04860, Bcin01g08050,*  *Bcin13g03880, Bcin04g01870,*  *Bcin04g03360, Bcin03g01040,*  *Bcin11g06270, Bcin09g03710,*  *Bcin01g05550, Bcin01g06935* |
| map00670 | One carbon pool by folate | 0.13920822 | 7 | *Bcin11g04890, Bcin02g08600,*  *Bcin01g09580, Bcin11g04890,*  *Bcin15g01620, Bcin07g06360,*  *Bcin12g02550* |
| map00230 | Purine metabolism | 0.17047456 | 14 | *Bcin06g00790, Bcin04g05060,*  *Bcin05g05580, Bcin09g03710,*  *Bcin03g05340, Bcin02g01760,*  *Bcin07g01690, Bcin13g03880, Bcin01g09580, Bcin12g00190, Bcin05g08280, Bcin03g06050, Bcin03g00860, Bcin15g02590,* |
| map00062 | Fatty acid elongation | 0.2506852 | 4 | *Bcin03g05840, Bcin02g04240,*  *Bcin15g05550, Bcin11g01860,* |
| map03430 | Mismatch repair | 0.30139858 | 7 | *Bcin01g04490, Bcin02g08740,*  *Bcin03g04190, Bcin14g01800,*  *Bcin02g00920, Bcin02g08740,*  *Bcin02g08740* |
| map00100 | Steroid biosynthesis | 0.33036531 | 8 | *Bcin02g00670, Bcin08g01540,*  *Bcin11g04690, Bcin01g00360, Bcin01g00350, Bcin16g04070, Bcin08g02190, Bcin04g01080* |
| map00300 | Lysine biosynthesis | 0.3366277 | 4 | *Bcin11g00440, Bcin01g01820,*  *Bcin14g05400, Bcin14g00430* |
| map00780 | Biotin metabolism | 0.3366277 | 4 | *Bcin03g08100, Bcin11g01810,*  *Bcin05g05450, Bcin14g04540* |
| map00260 | Glycine, serine and threonine metabolism | 0.33801354 | 10 | *Bcin03g06180, Bcin01g00210,*  *Bcin16g03500, Bcin04g03060,*  *Bcin02g08600, Bcin11g04890,*  *Bcin13g05020, Bcin03g05350,*  *Bcin11g00440, Bcin01g01820* |
| map00660 | C5-Branched dibasic acid metabolism | 0.34171312 | 2 | *Bcin04g02200, Bcin10g05310* |
| map00330 | Arginine and proline metabolism | 0.34207845 | 11 | *Bcin11g06270, Bcin04g00260,*  *Bcin05g08050, Bcin04g03360,*  *Bcin02g03180, Bcin09g03430,*  *Bcin13g05810, Bcin02g05730, Bcin07g00340, Bcin13g00080, Bcin01g06600* |

**Table S5.** DEGs for valine, leucine and isoleucine biosynthesis

| Gene _ id | Gene name | Log2FoldChange | p-value |
| --- | --- | --- | --- |
| *Bcin10g05310* | *Bcleu2* | 1.16926370287 | 0.00141700405172 |
| *Bcin01g00210* | *Bcilv1* | 2.56759945209 | 9.90764400889E-16 |
| *Bcin04g02200* | *Bcilv6* | 1.20340381627 | 5.40562584158E-5 |
| *Bcin04g03100* | *Bcilv5* | 1.55966041929 | 0.000663728512573 |
| *Bcin16g02700* | *-* | -1.05416560514 | 0.00654391754221 |
| *Bcin16g02820* | *-* | 1.34633708102 | 0.000262760137656 |
| *Bcin04g01520* | *-* | 1.44694309852 | 0.00123072268711 |

**Table S6.** DEGs for valine, leucine and isoleucine degradation

| Gene _ id | Gene name | Log2FoldChange | p-value |
| --- | --- | --- | --- |
| *Bcin04g01520* | *-* | 1.44694309852 | 0.00123072268711 |
| *Bcin13g01430* | *-* | 1.14015856977 | 0.000409335005756 |
| *Bcin13g03020* | *-* | 1.15837829032 | 0.000134800836042 |
| *Bcin11g04250* | *-* | 1.04324482663 | 0.00120731732042 |
| *Bcin16g03180* | *-* | 1.43456632037 | 2.13400044443E-5 |
| *Bcin03g05840* | *-* | 1.00700271079 | 0.00439189504325 |
| *Bcin08g05100* | *-* | 1.1299395507 | 0.000442631755412 |
| *Bcin08g05040* | *-* | 1.1500068863 | 0.000636792818984 |
| *Bcin08g03170* | *Bcehd3* | 1.1168667677 | 0.00470617742494 |
| *Bcin12g03570* | *-* | 1.00762956848 | 0.00541713241957 |
| *Bcin11g00330* | *Bcerg13* | 1.42032327777 | 0.000152474877916 |
| *Bcin13g05810* | *-* | 1.48156760476 | 2.10226443441E-5 |

**Table S7.** DEGs for alanine, aspartate and glutamate metabolism

| Gene _ id | Gene name | Log2FoldChange | p-value |
| --- | --- | --- | --- |
| *Bcin12g04860* | *-* | 1.52885278958 | 0.000660505532823 |
| *Bcin01g08050* | *-* | 2.2477473449 | 1.54105100441E-12 |
| *Bcin13g03880* | *Bcade13* | 1.83767365322 | 0.000238221110075 |
| *Bcin04g01870* | *Bcarg4* | 1.32623813303 | 5.56095215736E-5 |
| *Bcin04g03360* | *-* | 1.67653837768 | 3.12162393081E-5 |
| *Bcin03g01040* | *-* | 1.21293858108 | 0.00179085260922 |
| *Bcin11g06270* | *-* | 1.25493640011 | 0.00554757288342 |
| *Bcin09g03710* | *Bcade4* | 1.82368802853 | 0.00126025891513 |
| *Bcin01g05550* | *Bccpa2* | 1.91457051932 | 6.47143858514E-8 |
| *Bcin01g06935* | *-* | 1.33258798015 | 0.00247399832161 |

**Table S8.** DEGs for lysine biosynthesis

| Gene _ id | Gene name | Log2FoldChange | p-value |
| --- | --- | --- | --- |
| *Bcin11g00440* | *Bchom6* | 1.01148143542 | 0.00117238104582 |
| *Bcin01g01820* | *Bchom2* | 1.11894283069 | 0.00213866849759 |
| *Bcin14g05400* | *-* | 1.02430708067 | 0.0036784328735 |
| *Bcin14g00430* | *Bclys4* | 2.1473272069 | 3.19134032574E-7 |

**Table S9.** DEGs for glycine, serine and threonine metabolism

| Gene _ id | Gene name | Log2FoldChange | p-value |
| --- | --- | --- | --- |
| *Bcin03g06180* | *-* | 1.00761547788 | 0.00469244247542 |
| *Bcin01g00210* | *Bcilv1* | 2.56759945209 | 9.90764400889E-16 |
| *Bcin04g03060* | *Bctrp5* | 1.21384446341 | 0.00160592749886 |
| *Bcin16g03500* | *Bccys4* | 1.37184464632 | 0.00398343705227 |
| *Bcin02g08600* | *Bcshm2* | 1.16179593885 | 0.00622305394625 |
| *Bcin11g04890* | *-* | 1.36956697856 | 0.000156942156783 |
| *Bcin13g05020* | *Bcthr4* | 1.06269903174 | 0.000717573324259 |
| *Bcin03g05350* | *Bcthr1* | 1.3202861419 | 0.000109750650261 |
| *Bcin11g00440* | *Bchom6* | 1.01148143542 | 0.00117238104582 |
| *Bcin01g01820* | *Bchom2* | 1.11894283069 | 0.00213866849759 |

**Table S10.** DEGs for arginine and proline metabolism

| Gene _ id | Gene name | Log2FoldChange | p-value |
| --- | --- | --- | --- |
| *Bcin11g06270* | *-* | 1.25493640011 | 0.00554757288342 |
| *Bcin04g00260* | *Bcpro2* | 1.61976089547 | 0.000162852473294 |
| *Bcin05g08050* | *-* | 1.46341765059 | 7.46981098001E-6 |
| *Bcin04g03360* | *-* | 1.67653837768 | 3.12162393081E-5 |
| *Bcin07g00340* | *Bccar1* | -1.86510077426 | 5.05095923394E-5 |
| *Bcin13g00080* | *-* | -1.96465861488 | 0.00250581348061 |
| *Bcin01g06600* | *-* | -2.82156548763 | 1.77026949047E-5 |
| *Bcin02g03180* | *Bcspe2* | 1.98211471453 | 2.08531838184E-7 |
| *Bcin13g05810* | *-* | 1.48156760476 | 2.10226443441E-5 |
| *Bcin09g03430* | *-* | 1.32576701534 | 0.00646923775339 |
| *Bcin02g05730* | *-* | -2.35438921258 | 0.00197012768873 |


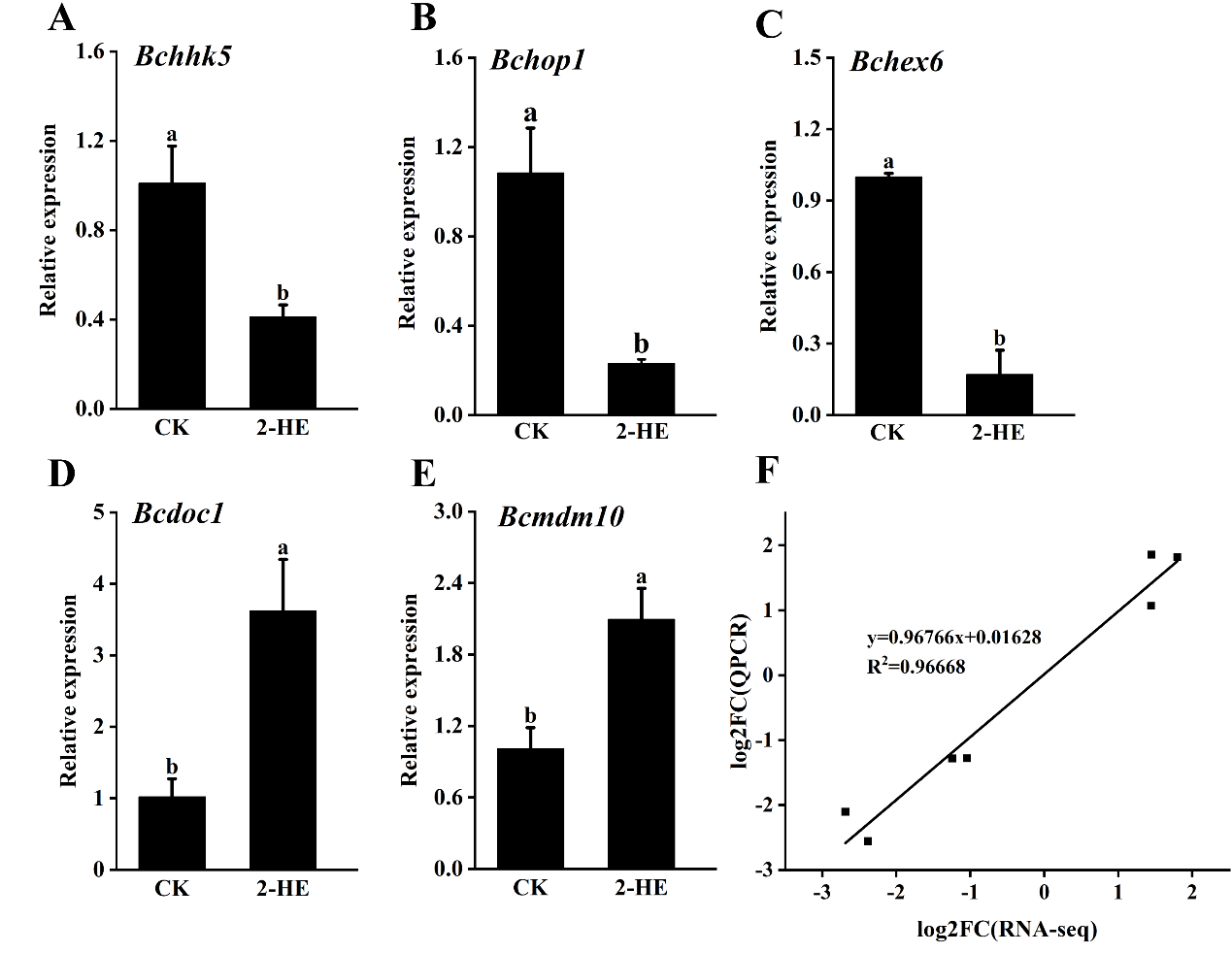
**Figure S1** DEGs was verified by qRT-PCR. (A - G) The DEGs relative expression level in untreated and 2-HE treated cells was detected by qRT-PCR. (H) Correlation analysis of DEGs expression levels and qRT-PCR in RNA-seq. Different lowercase letters indicate a significant difference between the 2-HE treatment group and the control group according to the independent sample T-test.
